# A Review on *CYP11A1*, *CYP17A1*, and *CYP19A1* Polymorphism Studies: Candidate Susceptibility Genes for Polycystic Ovary Syndrome (PCOS) and Infertility

**DOI:** 10.3390/genes13020302

**Published:** 2022-02-05

**Authors:** Roozbeh Heidarzadehpilehrood, Maryam Pirhoushiaran, Rasoul Abdollahzadeh, Malina Binti Osman, Maryam Sakinah, Norshariza Nordin, Habibah Abdul Hamid

**Affiliations:** 1 Department of Obstetrics & Gynaecology, Faculty of Medicine and Health Sciences, Universiti Putra Malaysia, Serdang 43400, Malaysia; roozbeh.heidarzadeh@gmail.com (R.H.); maryam.rom@upm.edu.my (M.S.); 2 Department of Medical Genetics, School of Medicine, Tehran University of Medical Sciences, Tehran 1417613151, Iran; maryam.pirhoushiaran@gmail.com (M.P.); rasoul142857@gmail.com (R.A.); 3 Department of Medical Microbiology, Faculty of Medicine and Health Sciences, Universiti Putra Malaysia, Serdang 43400, Malaysia; malinaosman@upm.edu.my; 4 Department of Biomedical Sciences, Faculty of Medicine and Health Sciences, Universiti Putra Malaysia, Serdang 43400, Malaysia

**Keywords:** genetic polymorphism, hyperandrogenism, infertility, steroidogenesis, PCOS

## Abstract

Polycystic ovary syndrome is a multifactorial condition associated with reproductive and endocrine organs and might cause infertility and metabolic abnormalities in childbearing age. PCOS seems to be a multifactorial disorder resulting from the combination of several genetic and environmental factors. Little research has been conducted to date on the impact of polymorphisms in infertility. We aim to review the appearance of polymorphisms in females of diverse ethnicities and their effect on infertility in the population with polycystic ovary syndrome. There have been numerous reports of the importance of the steroidogenesis pathway and genetic variants in PCOS pathogenesis. The most important genes that play a role in the aetiology of PCOS are *CYP11A1*, *CYP17A1*, and *CYP19A1*. We evaluated the occurrence of polymorphisms in various ethnicities in the *CYP11A1*, *CYP17A1*, and *CYP19A1* genes and their efficacy on increasing PCOS risk with infertility. Our findings revealed that polymorphisms in various ethnicities are associated with the risk of PCOS with infertility. Although conflicting results regarding *CYP11A1*, *CYP17A1*, and *CYP19A1* polymorphisms and their influence on PCOS with infertility have been reported in a small number of papers, the authors feel this may be attributable to the sample size and ethnic composition of the examined populations. In conclusion, our study strongly suggests that the *CYP11A1*, *CYP17A1*, and *CYP19A1* genes might significantly enhance the probability of developing PCOS with infertility.

## 1. Introduction

PCOS is a multifactorial disease caused by both genetic and environmental factors and leads to various symptoms [1]. Polycystic ovary morphology (PCOM), menstrual irregularities, and hyperandrogenism (HA) are the diagnostic features of females with PCOS [2]. This condition can be diagnosed based on the Rotterdam criteria or (NIH) National Institutes of Health consensus [3]. Female infertility, diabetes mellitus, hypertension, dyslipidaemia, obesity, and obstructive sleep apnoea are all increased risks for women who have polycystic ovarian syndrome [4]. Women with PCOS have an 11-fold increase in the prevalence of metabolic syndrome than age-matched controls, and these complications can eventually lead to cardiovascular disorders such as myocardial ischemia or infarction and an acute coronary syndrome that can be deadly [5]. In addition to infertility, women with PCOS also have an increased risk of gynaecological problems, including abnormal uterine bleeding and oligomenorrhea, which can ultimately cause uterine/endometrial cancer [6]. In addition, they are also at risk of developing psychological disturbances such as anxiety, depression, and eating disorders, which could be due to the physical manifestations of hyperandrogenism, such as hirsutism, male body configuration, and obesity [7,8]. The prevalence of PCOS varies, ranging from 4–20 percent depending on various subpopulations that are influenced by the environmental conditions and genetic variations [9]. Anovulation is one of the most significant reasons for infertility in females [10]. Women with PCOS commonly having anovulation with irregular menstrual cycles and sometimes with regular menses. PCOS intricates hormonal imbalances, mainly excessive androgen levels that possibly cause the anovulation [11]. Hyperandrogenism and anovulation have a critical aspect in infertile women with PCOS. Epidemiological data recommend that PCOS causes a high prevalence of ovarian dysfunction and anovulation, where both of these conditions are positively correlated with a progressive chance of infertility [12]. Women with polycystic ovary syndrome appear to experience various conditions during pregnancy, including abortion, preterm delivery, and intrauterine death or stillbirth [13,14].

PCOS is estimated to have a heritability of 70 percent however the genetic loci linked to it so far accounts for just 10 percent of that; on the other hand, increasing research suggests that altered epigenetic and developmental planning due to the hormonal imbalance of the uterine environment might play a role in developing PCOS [15,16]. Numerous studies on the effect of offspring susceptibility to metabolic diseases and obesity [17,18], as well as epigenetic modifications [19], such as DNA methylation, oxidative stress, and vascular diseases, on intergenerational and transgenerational inheritance have been conducted on non-human animals [20]. Overall, the evidence suggests that epigenetic changes occur during foetal life and may have an impact on the developmental origins of PCOS. This is based on the assumption that there is a critical time interval of foetal susceptibility beginning in early-to-mid-gestation when developmental programming occurs [21]. Moreover, researchers performed genome-wide DNA methylation profiling and RNA sequencing on ovarian tissue in both third-generation and control PCOS-like mice to determine their methylation patterns. The results demonstrate that the transfer of PCOS features to future generations happens via an altered environment of DNA methylation, and they suggest the use of methylome markers as a potential diagnostic landmark for the syndrome [22]. Animal models of prenatal testosterone treatment support clinical studies of PCOS women by inducing permanent PCOS-like phenotypes characterized by LH hypersecretion due to reduced steroid negative feedback, hyperandrogenism, ovulatory dysfunction, and impaired glucose–insulin homeostasis. These studies provide insights into when developmental programming occurs during human development, how placental function alters the maternal–foetal relationship to affect foetal growth, and when penetrance occurs. The improvement of the endocrine–metabolic health of PCOS women in order to eliminate their likelihood of gestation complications may have the ability to diminish intergenerational susceptibility to PCOS and the associated metabolic abnormalities in their offspring [23]. There is still much to learn about the intricate interplay of the genome, epigenome, and environmental variables. It is incredibly difficult to separate and trace the impact of each of these elements through generations, particularly in humans [24].

In addition to epigenetic studies, many studies have been conducted to decipher the genetic nature of PCOS. In addition, a number of studies have looked at the link between significant clinical aspects of PCOS and GWAS-identified variants in the last several years [25,26]. The goal of the PCOS GWAS loci analysis is to provide insight on the mechanism by which the risk of SNPs may impact the aetiology of PCOS status and, ultimately, to improve treatment outcomes. Following these studies, it appears that the genes DENND1A and TOX3, as well as the genes LHCGR, AMH, AMHR2, THADA, and INSR, are the most important genes in the susceptibility of PCOS. Genetic variations in the previously mentioned genes may be involved in several pathways, including insulin resistance, the dysregulation of androgen and gonadotrophins, and metabolic disorder-related pathways in PCOS pathogenesis [25,26,27]. There is ample evidence that polycystic ovary syndrome is an intrinsic syndrome, and in most of its clinical symptoms such as obesity and diabetes, traces of genetic variation might be seen [28]. The occurrence of alterations in the genes *HOXA-10* and *HOX-11* in women with polycystic ovary syndrome affects endometrial reception and might risk infertility with implantation failure [29,30]. Recent studies have shown that polymorphism in the *FSHR* gene is significantly associated with PCOS [31]. Even though the function of hereditary pathways in infertility is unequivocally accepted, genetic factors included within the aetiology of female infertility have not been thoroughly demonstrated presently. One of the most significant reasons for infertility with PCOS is the alteration and point mutation in genes involved in biochemical pathways, and one of the key pathways associated with infertility problems is steroidogenesis [32]. Steroidogenesis pathways represent an important topic to study because some genetic variants involved in hypothalamic-pituitary depletion and the gonadotropin receptor have been found to change in women with infertility issues. These changes can interfere with pathways such as androgens and ovulation, which may cause hyperandrogenism and ovulatory dysfunction, making them susceptible candidates worth examining. It is imperative to understand the genes involved in the steroidogenesis pathway. This review summarises and discusses the recent investigation of genetic pathways that have been associated with infertility among PCOS patients. We have chosen the genetic polymorphism of the genes involved in the steroidogenesis pathway as the candidate genetic variants that are susceptible to infertility between females with PCOS, focusing on *CYP11A1*, *CYP17A1*, and *CYP19A1*.

## 2. Biochemical Pathways in PCOS

Due to the polygenic nature of polycystic ovary syndrome, numerous biochemical pathways with various genes have been identified. Many studies have shown the impairment of androgen and ovulation pathways in women with PCOS, resulting in hyperandrogenism and ovulatory dysfunction. Among these biochemical pathways are genes associated with PCOS, including insulin secretion (e.g., *INSR*, *IRS*-1, *INS*), chronic inflammation (e.g., TNF-α, IL-6), coagulation cascade and complement (e.g., *VWF*), signalling (e.g., *AMH*, *LHCGR*, *INS*, *ADIPOQ*), cancer (e.g., *MMP*, *AR1*, *INS*), and steroidogenesis (e.g., *CYP11A1*, *CYP17A1*, *CYP19A1*) pathways [33,34].

## 3. Ovarian Steroidogenesis

**Figure 1:**
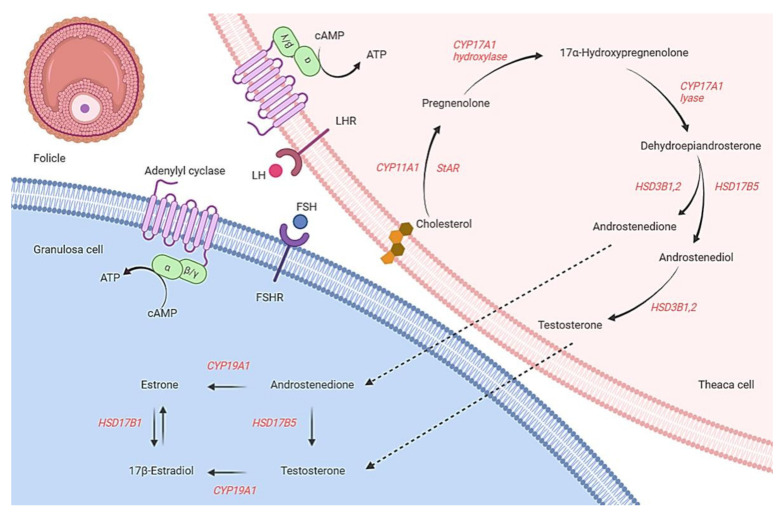
The red colours represent relevant genes in the female ovaries’ steroidogenesis process; cholesterol in the theca and granule cells’ membranes is the first precursor to convert to pregnenolone. One of the most significant processes for the production of sex hormones in women is steroidogenesis, which occurs in the theca and granulosa organs. Theca cells synthesise the required sex steroid hormones and use steroid enzymes to convert cholesterol to 17-b estradiol. LH—luteinizing hormone; *StAR*—steroidogenic acute regulatory protein; *CYP11A1*—cytochrome P450 11A1 (cholesterol side-chain cleavage enzyme); FSH—follicle-stimulating hormone; *CYP19A1*—cytochrome P450 19A1 (aromatase).

Steroidogenesis is a sequential process that converts cholesterol to bioactive compounds in steroidogenic-specific tissues under the control of certain steroidogenic enzymes. The adrenal cortex and the ovary are two examples of steroidogenic specialized organs that produce hormones that govern a variety of reproductive, endocrine, and metabolic activities in women, as well as fertility maintenance. As a result, a disruption in steroidogenesis has been linked to a variety of disorders, including polycystic ovarian syndrome. Therefore, understanding such disorders may provide essential insights for advances in infertility treatment among women with PCOS. Steroid hormones biosynthesis, including mineralocorticoids, progestins, androgens, oestrogen, and glucocorticoids, are conducted by the enzymes that are known as steroidogenic enzymes that constitute steroid reductases hydroxysteroid dehydrogenases (HSDs) and particular cytochrome P450 enzymes (*CYPs*) [35]. Steroidogenesis in the ovary starts with cholesterol modification and progresses sequentially to progestin, androgen, and oestrogen, all of which are required for subsequent processes synthesizing steroid hormones. These hormones are subsequently delivered into the blood circulation, where they exert their effects on both the peripheral (PNS) and central nervous systems (CNS) [36]. The synthesis of steroid hormones and the maturation of oocytes promote the maintenance of female reproductive tissues [37]. In the ovary, under the influence of the luteinising hormone (LH), cholesterol is converted to pregnenolone by *CYP11A1*, once bound to the interior layer of mitochondria in theca cells [23]. The *CYP17A1* gene converts pregnenolone to 17 hydroxypregnenolone and 17 hydroxypregnenolone to dehydroepiandrosterone (DHEA) as a result of androgen amalgamation captured within the thecal cells containing receptors to LH [24]. Follicle-stimulating hormone (FSH) is required for the *CYP19A1* aromatase to produce oestrogen in the granulosa cells. Oestrogens have a crucial part in controlling the ovary’s function. The mechanism of action of ovarian steroidogenesis is shown in Figure 1. Understanding these steroidogenesis pathways inside the ovary may give key information on the essential molecular regulators that regulate the signalling mechanism in ovarian cells, most notably in hyperandrogenism, ovulation, and fertility.

## 4. *CYP* Genes and PCOS with Infertility

Numerous studies have confirmed that hyperandrogenism is one of the most prominent clinical features reported in patients with polycystic ovary syndrome associated with heterogeneous phenotypes with diverse genetic variants. These situations, which involves an enzyme deficiency in the steroidogenesis pathway, is considered a prognosis for PCOS [38]. A subset of the *CYP* genes encodes enzymes involved in the process of steroidogenesis biosynthesis. Among them, the majority of research has focused on the *CYP19A1*, *CYP17A1*, and *CYP11A1* genes [39]. The *CYP19A1* gene encodes an enzyme that is a member of the cytochrome P450 family. The cytochrome P450 family of enzymes is involved in the synthesis of lipids, steroids, cholesterol, and the metabolism of drugs. Cytochrome P450 protein is located on the endoplasmic reticulum network, and catalysis is the final stage of steroid biosynthesis. Mutation in this gene might reduce or increase aromatase activity. The evaluation of phenotypes associated with abnormalities indicates the ambivalent function of oestrogens in growth and differentiation, as well as a sex steroid hormone [40]. The *CYP17A1* gene, which encodes the cytochrome P450 protein, catalyses multiple reactions including the production of steroidogenesis, androgens, glucocorticoids, and progestins. Mutations in this gene are associated with adrenal hyperplasia, pseudohermaphroditism, 17,20 lyase deficiency, and 17 α-hydroxylase deficiency [41]. The *CYP11A1* gene, which encodes a member of the cytochrome P450 family, is a protein located in the inner membrane of the mitochondria that catalyses the first step of steroid hormones and converts cholesterol to pregnenolone [42].

### 4.1. CYP11A1 Gene

*CYP11A1* gene is a subset of enzymes belonging to the cytochrome P450 superfamily, located on chromosome 15q24.1. The expression of these monooxygenases in the inner membrane of mitochondrial consequently plays a key role in steroid synthesis reactions, cholesterol, and drug metabolism. One of the vital roles is the catalysis of cholesterol to pregnenolone, which is the first and controlling step in synthesising steroid hormones. In addition, two isoforms, A and B, are encoded by two transcripts of variants of this gene [43]. The *CYP11A1* gene is expressed in various organs and tissues, including the ovaries, kidneys, breasts, testes, and bladder that cause various disease. Here, we intend to examine the occurrence of polymorphisms in the *CYP11A1* gene that increases PCOS risk with infertility. The effect of the *CYP11A1* 5’UTR sequence stability on the regulation of the gene as a locus for PCOS expansion, particularly in the development of hyperandrogenaemia, was further validated by a case–control study of 20 PCOS families from a British population. Gharani et al. was the first to establish a relationship between PCOS and the *CYP11A1* gene. The function of the *CYP11A1* gene is critical for the activity of the enzymes involved in androgen metabolism and cholesterol side-chain cleavage. The study discovered a significant correlation between polymorphisms in the pentanucleotide (*TTTTA*) repeat region of the *CYP11A1* 5’UTR and a high level of total serum testosterone in PCOS women [44]. The role of *CYP11A1* alleles in PCOS with hyperandrogenaemia was also investigated in the Greek population. A total of 170 women, consisting of 80 with PCOS and 90 healthy Greek women with regular menstrual cycles (<35 days), were studied. All the females selected were Caucasian and had registered consecutively over the past three years at the Endocrine Section of the First Department of Medicine, Athens University School of Medicine, Laiko Athens General Hospital. The study searched for alterations in the alleles for *CYP11A1 (TTTA)n* microsatellite (–528 pairs) in Greek females who complied with PCOS and ethnic controls, and the study showed a strong association between the allele and severity of hyperandrogenaemia in patients with PCOS [45]. Additionally, 100 cases of polycystic ovarian syndrome were evaluated in a study conducted on the population of Indian women. Increased testosterone levels were associated with the presence of six-repeat alleles in the *CYP11A1* gene [46]. A polymorphism analysis was carried out by Zhang and colleagues to estimate the association of *CYP11A1* SNP rs4077582 with PCOS susceptibility. A potent co-relationship between rs4077582 in *CYP11A1* and PCOS susceptibility was observed. In addition, an increase in androgen production by an alteration in the *CYP11A1* nucleotide was found, and this effect was confirmed by the alteration of testosterone levels in different genotypes. The study observed that the mRNA’s abundance was due to the *CYP11A1* promoter’s increased activity, which was differentially controlled by the sequences of the 5’UTRs of the mRNA [47]. Likewise, in a similar case study, a population of Egyptian women with polycystic ovary syndrome, 53 patients and 53 controls in the rs4077582, were evaluated, which positively supported the occurrence of polymorphism and its association with PCOS [48]. In some research performed on the *CYP11A1* promoter, It has been proposed that the regulation of the *CYP11A1* promoter in granulosa cells is controlled by liver receptor homologue-1 (LRH-1) and Steroidogenic element-1 (SF-1) [49]. A 1999 study also supported this hypothesis, and it showed the loss of P450scc due to the knockout SF-1 [50]. Because increases or decreases in the LH hormone have an effect on the downstream responses of steroid hormones in people with various rs4077582 genotypes, the relationship between *CYP11A1* and gonadotropins is not clear. In addition, a study conducted on a population of Indian women, among 267 cases diagnosed with polycystic ovary syndrome in comparison to 275 healthy controls, the *CYP11A1* polymorphic pentanucleotide repeat was analysed by the PCR-PAGE technique, which showed that the increasing recurrence of polymorphisms could be a biomarker for the risk of PCOS. One of the most important indicators of this study was the study of recurrences in both control and patient groups. These copies ranged from a minimum of 2 to a maximum of 16 repetitions, and the examination of demographic data revealed that individuals who showed recurrences of more than 8 had symptoms of ovarian polycystic ovary syndrome. Recurrences of less than eight were obtained in the control group [51]. The results of this study were consistent with the results of a study conducted on Grecian women [45]. This might be assumed that genetic polymorphisms in the *CYP11A1* prompter may influence gene expression and may result in a condition related to hormonal changes, such as polycystic ovarian syndrome. Recently, in a case–control study of 128 Iraqi women with PCOS, the incidence of pentanucleotide recurrence was found to be significantly associated with polycystic ovary syndrome. The most significant finding of this study was the prevalence of five and three repeats in the *CYP11A1* promoter in Iraqi women, who reported 62 percent and 79.7 percent of the total repeats in *CYP11A1*, respectively [52], compared to pentanucleotides with nine repeats among the population of Caucasian women in America, six and eight repetitions in Chinese women [53], and four repeats in white women in Spain [54].

In contrast to the research records discussed so far, no significant relationship has been reported in a number of studies that can be cited in Hispanic white women [55], Chinese women [56,57], Argentinian women [58], and Czech women [59]. A recent meta-analysis study reviewed some of the previously reviewed studies, which confirmed a strong association between the occurrence of a recessive trait in individuals and the occurrence of 4R-pentanucleotide recurrences, even as companies of 6R-p confirmed the reduced risk of PCOS considering the dominant trait [60]. In addition, another meta-analysis confirmed a strong correlation between polymorphism recurrences in *CYP11A1* in the white female population [61], and some studies reported a correlation between these recurrences and an increase or decrease in testosterone levels. In some of these reports, carriers of shorter alleles show higher serum testosterone levels [46]. In other studies, however, there was no association between increased androgen changes in polymorphisms and changes in *CYP11A1* transcription [56,62]. In addition, these alleles’ variation has a correlation with metabolic traits and obesity [55], a reduction in blood lipids [59], decreased FSH levels [59], and waist to hip ratio, Lower glucose [57] also show a significant correlation incidence with rs11632698 polymorphism, and increased [57] and decreased [47] risk of PCOS in Chinese women rs4077582, and its association with the development of polycystic ovary syndrome and changes in the levels of LH and testosterone [47]. Based on the case history discussed so far (Table 1), *CYP11A1* might be considered as a biomarker, which is most likely correlated with infertility in polycystic ovary syndrome.

### 4.2. CYP17A1 Gene

The *CYP17A1* gene located on chromosome 10q24.3. It is a subset of enzymes belonging to the cytochrome P450 superfamily. These monooxygenases are expressed in the endoplasmic reticulum and hence play a critical role in steroid production reactions, cholesterol metabolism, and drug metabolism [63]. Enzyme 17-a-hydroxylase/17–20 lyase is encoded by the *CYP17A1* gene and converts pregnenolone into 17-hydroxypregnenolone, and 17-hydroxypregnenolone is converted to dehydroepiandrosterone (DHEA) and 4-androstenedione via 17,20-lyase activity. The *CYP17A1* gene is expressed in various tissues, including the gonads and adrenal cortex, where defects cause various diseases. Here, we study the incidence of polymorphisms in the *CYP17A1* gene that causes robust PCOS risk with infertility [64]. One of the significant factor genes in the expansion of PCOS is the *CYP17A1* gene. In 2007, B. Echiburú et al. investigated *CYP17A1* promoter polymorphisms in the Chilean female population; a total of 159 objects with PCOS manifestation were examined. The study discovered that the rs743572 T > C polymorphism in the *CYP17A1* gene was one of the variations associated with an increased risk of PCOS. Additionally, clinical and hormonal confirmation was accomplished by the evaluation of insulin tolerance and body weight. A change in the *CYP17A1* gene, as well as the presence of the rs743572 T > C polymorphism in this gene, are associated with elevated lipid levels, glucose resistance, and weight gain. As a result, polymorphisms in the *CYP17A1* gene can be associated with PCOS, along with metabolic pathways [65]. A study was conducted in 2008, and it was performed among 200 Indian women. In this study, the main conclusion is based on data from nucleotide sequencing at the site of *CYP17A1* polymorphisms, as well as the study of the hormones including dehydroepiandrosterone DHEAS, 17a-hydroxyprogesterone, androstenedione, and serum testosterone levels to evaluate the 152bp region inside the promoter sequences of *CYP17A1* in a distinct combination of women with PCOS manifestations based on the Rotterdam criteria. The promoters of the *CYP17A1* gene were evaluated as this region controls the enzymes that regulate the rate-limiting steps in androgen synthesis. The finding of this study revealed an independent association between the polymorphisms of the promoters and the hyperandrogenism found in PCOS patients [46].

In a 2015 case–control study of Iraqi women, two groups of individuals were examined for genetic association between the *CYP17A1* allele and the incidence of polycystic ovary syndrome. Out of 123 participants, 84 were confirmed by clinicians with hormonal and clinical manifestations of polycystic ovary syndrome. They were also measured to evaluate the hormones testosterone, prolactin, FSH, and LH. Finally, by the genotypic analysis of individuals, three genotypes were reported, including the most common allele among patients, the C. allele. The TC genotype was the most common allele among controls, and the TT allele was a wild homozygous allele. In addition, as a finding, to the amount of the CC genotype in the population of women with polycystic ovary syndrome and its absence in healthy individuals, the importance of –34 (T/C) polymorphism might be considered as a marker to identify the disease [66]. In the same population, another study was conducted in 2014 by Marwa B. M et al.; on the Iraqi women population: 2 groups of 61 women with polycystic ovary syndrome and 30 in the control group were analysed for genotype analysis of the effect of polymorphism and its relationship with the disease. Between the control and patient groups, there were significant differences in the FSH and HDL levels; however, there were no significant differences in the cholesterol, triglyceride, VDL, or LDL levels. In addition to genotyping, TC was reported as the mutant allele and TT as the wild allele; nevertheless, the genotyping investigation did not reveal a relationship between polycystic ovarian syndrome and the *CYP17A1* polymorphism [67].

In another study conducted in 2018 on 250 cases of polycystic ovary syndrome and 250 cases in the North Indian female population as a control, the rs743572 allele was analysed in *CYP17A1.* For the biochemical evaluation of patients, the hormones HDL, triglyceride, and cholesterol were analysed in both groups of patients and the control group. The results of the polymorphism study at –34 T/C in *CYP17A1* revealed a significant difference in the group of women with polycystic ovary syndrome compared to the control group and similarly confirmed the association of the rs743572 allele in women in northern India with PCOS [68] with a study conducted in the Greek population [69]. In 2017, the case–control study by Z. Rahimi and E. Mohammadi was performed among the women population of western Iran. One hundred and nine individuals were enrolled as controls against fifty women with PCOS after clinical examinations and confirmed by a gynaecologist. The aim of this research was to investigate the polymorphism in -34 T/C in *CYP17A1*. In addition, for clinical evaluation of patients, measurements of various hormone levels were taken, including sex hormone-binding globules, dehydroepiandrosterone (DHEA) and oestradiol. The results of the essays revealed that the levels of SHBG and oestradiol in the group of patients with polycystic ovary syndrome were lower than the control group. In contrast, the level of DHEA in patients was higher than in the control group. In addition, a positive association was reported between the incidence of symptoms of polycystic ovary syndrome in patients and carriers of polymorphism in –34 T/C [70]. In a recent study of the Pakistani female population in 2020, 234 women with polycystic ovary syndrome with two phenotypes of three prototypes based on ASRM consensus were among the 344 participants in the project. In this study, the SNP rs743572 was amplified in *CYP17A1* by the PCR-RFLP method. The findings of this study demonstrated unequivocally that the frequency distribution of the TC genotype is greater in patients with PCOS than in controls. In Pakistani women, there was a substantial correlation between the risk of infertility in polycystic ovary syndrome and the occurrence of the *CYP17*A1 polymorphism [71]. Another case study was conducted by S. Ashraf et al. on the Kashmir women population in India in 2020. The aim of this study was to investigate the relationship between hyperandrogenism and PCOS; for this purpose, they examined the number of t394 patients with PCOS against 306 control samples. To evaluate the relationship between *CYP17A1* and their hyperandrogenism, they measured hormonal and biochemical tests and anthropometer measurements between the case and control groups. In addition, in order to analyse the genotype, PCR-RFLP was applied. The results of their study showed that between the distribution of alleles, there was no significant difference in genotype between case and control individuals, while there is a significant relationship between increased testosterone levels and clinical phenotypes such as alopecia between patients and healthy individuals [72]. In 2018, L. Wang studied 17 subfamilies in the Chinese female population, in which he found a link between polycystic ovary syndrome and insulin resistance and blood pressure. The purpose of this case–control study, which included 1,860 individuals from the control and case groups, was to determine the impact of *CYP17A1* polymorphism on the control and patient groups. Their research established a strong association between the rs2413409, rs17115149, and rs10044677 alleles with the occurrence of type 2 diabetes mellitus. The conclusion of their study established an association between the rs17115149 allele and the case group [73]. Contrary to the results stated above, a number of studies also have differences in results and found no association between the presence of polymorphisms in the *CYP17A1* –34T/C allele and hyperandrogenism in PCOS patients, such as in the study of Thai women population in 2015 by Ka. Techatraisak [74], in Turkey [75], and Caucasians in America [76]. A large number of polymorphism studies in different ethnicities and in different parts of the world have analysed the relationship between *CYP17A1* and PCOS [77]. The description and identification of polymorphisms in *CYP17A1* three-nucleotide variants was investigated for the first time by Crocitto et al. [78]. A report of the transition between a base pair of cytosine to adenine at nucleotide 5144 in the *CYP17A1* gene was made by Miyoshi et al. [79]. There are studies in which a significant association between SNP – 34T/C in *CYP17A1* and PCOS has been reported, which has been confirmed by researchers in various ethnicities, including Indians [68], Grecians [69], and Koreans [80]. Allele –34 in *CYP17A1* has also been reported as a valuable biomarker in assessing breast cancer in females [81] and in males [82,83]. In addition, it is probably associated as a biomarker associated with prostate cancer in men [84]. Even though these studies may seem to suggest that *CYP17A1* is not directly the susceptibility gene that causes infertility, the abnormal activity of the gene may have an effect on the sequential reactions of the steroidogenesis pathways in which, together with other defective genes in the pathway, may contribute to infertility in PCOS women. Table 2 describes the types of genetic variants associated with infertility in women with polycystic ovary syndrome as mentioned earlier.

### 4.3. CYP19A1 Gene

One of the crucial enzymes in oestrogen synthesis is aromatase, an enzyme encoded by the cytochrome P450 family 19 subfamilies A member one, *CYP19A1* gene. The gene is located on the short arm of chromosome 15 (15q21.2) and is expressed in some of the vital organs, including ovaries, testicular, adipose, bone, placental, and cerebrum tissues. In females, aromatase is most active in the ovaries. Mutations of the *CYP19A1* gene have been associated with various aromatase deficiency characterized by a low oestrogen level and a high level of androgen [86]. The activity of the gene is controlled by nine consecutive exons positioned in the long 93 kb gene regulative factor. In the ovary, the aromatase enzyme production is organized by a portion of the promoter located 1 kb pair upstream of exon two of the gene [87]. Irregular activation of the promoter could cause oestrogen-stimulating disorders, including breast cancer and endometriosis. Here, we aim to investigate the incident of polymorphisms in the *CYP19A1* gene that increases PCOS risk with infertility. A high number of polymorphisms of 7 < *n* < 13 repeats in intron number 4 has been associated with a high level of aromatase concentration. An aggregation of incomplete development of follicles explains PCOS because of the decreasing densities of positional oestrogen and aromatase performance [88]. The alteration of 10 or 12 repeats in the genetics’ allele has been proposed as a potential breast cancer allele associated with hyperactive aromatase enzyme activity. Women with PCOS, on the other hand, had shorter *CYP19A1* alleles with more than nine *(TTTA)n* repeats. Notably, these individuals with PCOS had the greatest blood testosterone and testosterone/oestradiol ratios during the early follicular phase of the menstrual cycle [89]. Studies on the *CYP19A1* gene also identified its different variants associated with the risk of developing reproductive cancers in women. Hence, further investigation of the *CYP19A1* gene polymorphisms may be the subject of further research. Alterations in the *CYP19A1* gene polymorphisms may affect the aromatase enzyme activity by acting on it; therefore, the results obtained in these changes and their effects on folliculogenesis regulation and induction of ovulation can be used in the treatment of infertility. Additionally, the results of polymorphism studies may aid in predicting the likelihood of a favourable response to in vitro fertilisation (IVF) infertility therapy. As a result, miscarriage risk is decreased, and fertility is likely to rise [90]. These findings are in line with those found in the literature, which, in 2010, Lee et al. screened for *CYP19A1* SNPs among Koreans. The *CYP19A1* gene was directly sequenced from 50 normal cases. A sum of 19 variations was identified: 4 in exons, 10 in introns, 6 in the 5′-untranslated regions (UTR), and 1 in 3′-UTR. The distribution of *CYP19A1 (TTTA)n* polymorphisms found was such that the most frequent allele was *(TTTA)*7 (66 percent), followed by *(TTTA)*11 (30 percent), *(TTTA)*12 (3 percent), and *(TTTA)*13 (1 percent). They resequenced the *CYP19A1* gene for the first time in a Korean population and discovered that these variants have an essential function in the oestrogen pathway [91].

Many studies have revealed that such alterations in the oestrogen production rate may lead to the prospect of oestrogen-related diseases, such as breast, endometrial, and prostate cancers. Moreover, cancer studies have revealed that *CYP19A1* also plays a role in obesity and infertility in Chinese women [92]. A case–control study was undertaken in 2014 on 225 normal Chinese women and 146 patients with endometriosis-related infertility, as proven by pathological and laparoscopic criteria. On the other hand, they discovered that the AA genotype of the *CYP19A1* polymorphism was strongly associated with an increased risk of endometriosis and infertility in the population [93]. In a case–control study conducted in 2011 on 600 Greek women, the case group was treated with gonadotropin stimuli. The aim of this study was to investigate the presence of polymorphism *(TTTA)n* in *CYP19A1* and its effect on treatment outcome. The results showed that there was a positive relationship between recurrences of *(TTTA)n* polymorphism and FSH hormone levels and the number and size of follicles in the subjects [94]. In another study conducted in another ethnicity in China. X. Zhang et al. examined 661 people, and the aim was to investigate the relationship between rs2470152 polymorphism and aromatase activity in the Chinese female population. The results of genotype studies revealed that the presence of rs2470152 polymorphism was not directly related to PCOS occurrence. However, in individuals with the TC genotype, the risk of hyperandrogenism and PCOS may increase due to the inhibition of aromatase enzyme activity [95]. Another study conducted in 2012 by L. Lazaros et al. examined the effect of the *CYP19A1* variant on infertility treatment in patients with the polycystic ovarian syndrome; in this study, in which 322 Greek women participated, a significant association between the occurrence of 7 repeats in *(TTTA)n*, *CYP19A1* polymorphism, and its effect on gonadotropin treatment was proven [96], This effect is most likely explained by a change in the ratio of androgen to oestrogen; nevertheless, these findings contradict those published by P. Xu et al. in 2013 among 522 cases and controls. The purpose of this study was to determine the link between the *(TTTA)n* allele polymorphism in *CYP19A1* and the risk of developing PCOS. In this research, fluorescent DNA fragments obtained from PCR confirmed the outcomes of testing different repeats, including 7, 8, 10, 11, and 16 repeats, and no significant relationship was observed between the incidence of the allele *(TTTA)n* in the Chinese female [97]. At the same time, another study was performed on Chinese women, and in this population, including 293 females, the aim was to evaluate the effect of rs700519, rs700518, and rs727479 alleles in patients treated with the ART method among PCOS patients. The results show that the CGT and CAT haplotypes were desirable to a fertility result in PCOS patients [98].

Another polymorphism study was performed in Iranian women and 140 people in this case–control study to ascertain a link between rs2414096 *CYP19A1* and polycystic ovary syndrome; outcomes revealed that the rs2414096 variant was significantly involved in the probability of individuals developing PCOS [99]. In 2018, a case and control study was conducted by R. Kaur et al. where 500 women from the North Indian population were analysed, and two alleles, rs2414096 and rs700519, were examined among the two groups of control cases. In addition, the PCR-RFLP method was employed to examine the genotype of individuals. The study results did not show a significant association in the study groups [68]. The studies of these alleles in different ethnicities in other countries have confirmed the entity of a relationship that indicates the impact of ethnicity and geography studied. China [100] was shown to be significantly associated with the age of menarche in Han Chinese women, FSH levels, with increased oestradiol to testosterone ratios (E2/T), and finally, increased risk of PCOS development. This argument is consistent with the findings of *n*. Xita in Greece [101].

The hormonal imbalance caused by anovulation and subsequent infertility in patients with polycystic ovary syndrome is associated with a loss of oestrogen production inhibition, and there is a significant association between increased oestrogen levels and the development of hyperplasia and, subsequently, an increased risk of endometrial cancer in women with PCOS [102]. Endometrial cancer is one of the oncoming cancers in developed countries and is also the fourth most common cancer in the world [103]. The most important risk factors for this cancer include polycystic ovary syndrome, diabetes, and obesity [104]. Conceptually similar work has also been carried out by A. Ayyob et al. on the Iraqi women population. Significant correlation was reported between the presence of polymorphisms in *CYP19A1* and endometrial cancer. In this study, which was performed employing 122 Iraqi women, a remarkable association was reported between the presence of alleles with longer (*n* ≥ 9) repetitions and the risk of endometrial and ovarian cancer. Repetitions of *(TTTA)n* 12, *(TTTA)n* 11, and *(TTTA)n* 9 were reported to be OR 1.56, 2.16, and 1.56, respectively, with the probability of doubling cancers’ risk [105]. This evidence is supported by the results of a systematic review conducted in 2016 [106]. The authors believe that the increased risk of endometrial cancer might be correlated with the increased prevalence of infertility in the population of women with polycystic ovary syndrome. Another pilot study was conducted among the Indian female population between 2018 and 2019, and the rs2470152 polymorphism was considered in *CYP19A1*. In this study, which employed 300 individuals, the results confirmed a correlation between the incidence of rs2470152 polymorphism and the risk of developing polycystic ovary syndrome [107]. These results are confirmed by evidence obtained by M. Rowaa et al. In this study, they examined the association of polymorphism in rs2414096 and the possibility of hyperandrogenism in the Egyptian female population. The results confirmed an increase in the ratio of LH to FSH in women with polycystic ovary syndrome. In addition, a significant relationship was reported between the incidence of rs2414096 polymorphism and the possibility of PCOS [108]. The implications of our findings might suggest that *CYP19A1* is probably the susceptible gene that contributes to infertility. Still, the abnormal transcripts may affect the sequential reactions of the steroidogenesis pathways which, together with other defective genes in the pathway, may contribute to infertility in PCOS women. Table 3 describes the types of *CYP19A1* genetic variants associated with infertility in women with polycystic ovary syndrome.

## 5. Concluding Remarks

Females worldwide are affected by polycystic ovary syndrome, a multifactorial endocrine condition that affects several systems, leading to infertility and anovulation [109]. Women with PCOS are more likely to develop type 2 diabetes mellitus (T2DM) as a result of the metabolic syndromes and cardiovascular complications associated with PCOS. In the majority of studies, changes in steroidogenesis pathways, notably in hormone synthesis and control, have been linked to the prevalence of PCOS. Women’s menstrual cycles are regulated by the natural functions of hormones. As a result, PCOS women may experience infertility as a result of interrupting these fertility cycle processes. It is possible that ovarian cysts are caused by long-term irregularities in women’s hormone levels. On the other hand, women with PCOS are more likely to have elevated levels of androgen, a masculine hormone [110].

Because of its multifactorial nature, both genetic and environmental factors are involved. Accordingly, identifying the susceptibility genetic variations that are key factors in modulating the production of steroids may give crucial insights into the development of new therapeutic options for PCOS and infertility concerns in women. Because each individual’s SNP profile is unique, more investigations in various populations are required to resolve the controversy surrounding the effect of *CYP11A1*, *CYP17A1*, and *CYP19A1* polymorphisms on infertility and PCOS. Although some of the CYP genes we were reviewing in this research have not been repeatedly associated with PCOS and infertility clinical manifestations, some of them have been repeatedly associated with previously mentioned disease symptoms in different populations: (1) *CYP17A1*: rs743572 in Chile [65], India [46,58,72], Iraq [67], Iran [70], and Pakistan [71]; (2) *CYP19A1:* tetranucleotide allele with 7 repeats *(TTTA)*7 in Greece [94,96], rs2470152 in China [95], and India [107], and rs2414096 in Iran [99], Egypt [109], and Iraq [66]. In summary, however, most research only covers a small portion of the subject of genetic variations. As a consequence, it is envisaged that future developments will result in a better understanding of the role of *CYP* genetic variations in the aetiology of PCOS with infertility.

## Figures and Tables

**Table 1:** Correlation between CYP11A1 genetic variants and PCOS with infertility.

Gene Name	SNP/STR Allele	Study Design	Country	Ethnicity	SS; Case/Control	Genotype Methods	Diagnostic Criteria	Clinical Characteristics	Risk Association	Ref
*CYP11A1*	*(TTTTA)n* *n* = 4 *, 6, 8, 9	Case–control study	Greece	Greek	80/90	PCR	NIH/NICHD	Hyperandrogenism	The pentanucleotides allele with 4 repeats (*n* = 4) is associated with higher serum total testosterone levels in PCOS.	[45]
*CYP11A1*	*(TTTTA)n* *n* = 4, 6 *, 8, 9, 10, 12	Case–control study	India	Asian	100/100	PCR	Rotterdam	Hyperandrogenism	A significant association of the pentanucleotides allele with 6 repeats (*n* = 6) with total testosterone levels is observed in PCOS group.	[46]
*CYP11A1*	rs4077582	Case–control study	Egypt	Egyptians	53/53	PCR–RFLP	Rotterdam	Diabetes mellitus, hirsutism, hyperandrogenism	The incidence of this polymorphism was positively associated with PCOS.	[48]
*CYP11A1*	*(TTTTA)n* *n* = 2–16, 8 *	Case–control study	India	Asian	267/275	PCR-PAGE	Rotterdam	Hyperandrogenism	There was a significant increase in the pentanucleotides allele with 8 repeats (*n* = 8) with higher testosterone levels in PCOS.	[51]
*CYP11A1*	(AAAAT)*n* *n* = 3, 5 *, 6, 7, 8	Case–control study	Iraq	Asian	74/58	PCR/sequencing	Rotterdam	Polycystic ovaries oligovulation, hyperandrogenism	There was a significant relationship between the pentanucleotides allele with 5 repeats (*n* = 5) and PCOS.	[52]
*CYP11A1*	*(TTTTA)n* *n* = 4, 6, 8, 9	Case–control study	Spain	Caucasian	92/33	PCR/ sequencing	NIH/NICHD	Hyperandrogenism Hirsutism	No association was found between *(TTTTA)n* in CYP11A1 and hyperandrogenism.	[55]
*CYP11A1*	*(TTTTA)n* *n* = 4, 6, 8, 9	Case–control study	China	Asian	96/78	PCR	Rotterdam	Hyperandrogenism	No association was found between *(TTTTA)n in CYP11A1* and hyperandrogenism.	[56]
*CYP11A1*	*(TTTTA)n* *n* = 4, 8	Case–control study	China	Asian	125/121	PCR	Rotterdam	Anovulation & Hyperandrogenism	There is no statistically significant difference.	[57]
*CYP11A1*	*(TTTTA)n* *n* = 4, 6, 8	Case–control study	Argentine	Non-Caucasian	65/58	PCR	NIH/NICHD	Hyperandrogenism	No statistically significant relationship was found between the pathogenesis of *CYP11A1* and PCOS.	[58]
*CYP11A1*	*(TTTTA)n* *n* = 4	Case–control study	Czech	Caucasian	256/109	PCR	Rotterdam	Hyperandrogenism	There is no statistically significant difference.	[59]

*n*: Number of Repeats; NIH/NICHD: National Institutes of Health/National Institute of Child Health and Human Development; Rotterdam: Rotterdam PCOS Consensus, * Risk alleles which is associated with clinical manifestation(s) of PCOS; SS: Sample Size.

**Table 2:** Correlation between CYP17A1 genetic variants and PCOS with infertility.

Gene Name	SNP/STR Allele	Study Design	Country	Ethnicity	SS; Case/Control	Genotype-Methods	Diagnostic Criteria	Clinical Characteristics	Risk Association	Ref
*CYP17A1*	rs743572	Case–control study	Chile	Caucasian	66/93	PCR -RFLP	NIH/NICHD	hormonal and clinical evidence of PCOS	*CYP17A1* is associated with metabolic pathway and obesity in women with PCOS	[65]
*CYP17A1*	rs743572	Case–control study	India	Asian	100/100	PCR-sequencing	Rotterdam	Anovulation	*CYP17A1* was associated with hyperandrogenaemia	[46]
*CYP17A1*	rs743572	Case–control study	Iraq	Asian	84/65	PCR	-	Anovulation	Positive correlation	[85]
*CYP17A1*	rs743572	Case–control study	Iraq	Asian	61/30	PCR-RFLP	Rotterdam	PCOS criteria	There is a correlation between the –34 T > C allele and increasing HDL	[67]
*CYP17A1*	rs743572	Case–control study	India	Asian	250/250	PCR-RFLP	Rotterdam	Anovulation	*CYP17A1* 34 T > C occurrence had a significant relation with PCOS patients	[68]
*CYP17A1*	rs743572	Case–control study	Iran	Asian	50/109	PCR-RFLP	Rotterdam	PCOS criteria	Polymorphism in *CYP17A1* is associated with the risk of PCOS	[70]
*CYP17A1*	rs743572	Case–control study	Pakistan	Asian	204/100	PCR-RFLP	Rotterdam	PCOS criteria	rs743572 is correlated with the occurrence of PCOS	[71]
*CYP17A1*	rs743572	Case–control study	India	Asian	394/306	PCR-RFLP	Rotterdam	Hyperandrogenism	Confirmation of the role of SNP in hyperandrogenism with increasing androgen	[72]
*CYP17A1*	rs1004467, rs17115149, rs12413409	Case–control study	China	Asian	440/1320	PCR-Genotype	-	Type two diabetes Mellitus	*CYP17A1* might be considered as a risk factor to T2DM	[73]
*CYP17A1*	rs743572	Cross-sectional study	Thailand	Asian	210	PCR-RFLP	Rotterdam	PCOS criteria	There was no significant association between –34 T > C and insulin resistance	[74]
*CYP17A1*	rs743572	Case–control study	Turkey	Asian	44/50	PCR-RFLP	Rotterdam	PCOS criteria	There was no association among case and control groups in allele –34 T > C	[75]
*CYP17A1*	rs743572	Case–control study	America	Caucasian	259/161	PCR	NIH/NICHD	PCOS criteria	There was no association with the development of PCOS and *CYP17A1* C/T	[76]

NIH/NICHD: National Institutes of Health/National Institute of Child Health and Human Development; Rotterdam: Rotterdam PCOS Consensus SS: Sample Size.

**Table 3:** Correlation between CYP19A1 genetic variants and PCOS with infertility.

Gene Name	SNP/STR Allele	Study Design	Country	Ethnicity	SS; Case/Control	Genotype Methods	Diagnostic Criteria	Clinical Characteristics	Risk Association	Ref
*CYP19A1*	*(TTTA)n* *n* = 7 *–12	Case–control study	Greece	Greek	300/300	PCR-PAGE	-	Male factor-infertility	There was a significant association between the tetranucleotide allele with 7 repeats (*n* = 7) and FSH level.	[94]
*CYP19A1*	rs2470152	Case–control study	China	Asian	364/297	PCR- RFLP	Rotterdam	PCOS features	rs2470152 polymorphism was associated with aromatase activity	[95]
*CYP19A1*	*(TTTA)n* *n* = 7 *–12	Case–control study	Greece	Greek	132/200	PCR-PAGE	NIH/NICHD	Ovulatory-dysfunction	Carriers with the tetranucleotide allele with 7 repeats (*n* = 7) presented higher testosterone levels compared to non-carriers with the tetranucleotide allele with 7 repeats (*n* = 7)	[96]
*CYP19A1*	*(TTTA)n* *n* = 7, 8, 10, 11, 12, 13	Case–control study	China	Asian	222/281	PCR-Capillary electrophoresis	Rotterdam	PCOS features	PCOS patients showed a higher frequency of short alleles compared to controls.	[97]
*CYP19A1*	rs727479 rs700518 rs700519	Case–control study	China	Asian	150/143	PCR-DHPLC	Rotterdam	PCOS features	The CGT and CAT haplotypes were desirable to fertility result in PCOS patients	[98]
*CYP19A1*	rs2414096	Case–control study	Iran	Asian	70/70	PCR-RFLP	Rotterdam	PCOS features	There was a positive relation between rs2414096 alleles in PCOS group	[99]
*CYP19A1*	rs700519 rs2414096	Case–control study	India	Asian	250/250	PCR-RFLP	Rotterdam	PCOS features	No significant association was found between alleles and PCOS	[68]
*CYP19A1*	rs2470152	Case–control study	India	Asian	120/180	PCR-RFLP	Rotterdam	PCOS feature	Allele rs2470152 was significantly higher in women with PCOS	[107]
*CYP19A1*	rs2236722	Case–control study	Iran	Asian	50/109	PCR	Rotterdam	Ovarian dysfunction	Incidence of rs2236722 polymorphism may not be associated with an increased risk of PCOS	[70]
*CYP19A1*	rs2414096	Case–control study	Egypt	Egyptians	30/30	PCR-RFLP	Rotterdam	PCOS criteria	Allele rs2414096 was associated with aromatase deficiency and hyperandrogenism	[109]
*CYP19A1*	rs2414096	Case–control study	Iraq	Asian	84/65	PCR-RFLP	Rotterdam	Oligomenorrhea	Allele rs2414096 present was related to hyperandrogenism in case group	[66]

DHPLC: Denaturing high-performance liquid chromatography; *n*: Number of Repeats; NIH/NICHD: National Institutes of Health/National Institute of Child Health and Human Development; Rotterdam: Rotterdam PCOS Consensus; * Risk alleles which is associated with clinical manifestation(s) of PCOS; SS: Sample Size.

## Data Availability

All the analysed information has been published in this article.

## Abbreviations

ADIPOQ	Adiponectin
ARI	Aldehyde reductase 1
AMH	Anti-Mullerian hormone
AIs	Aromatase inhibitors
CYP21	21-hydroxylase gene
CYP11A	Cholesterol side-chain cleavage enzyme
CYP19A1	Cytochrome P450 family 19 subfamilies A member 1
CYPs	Cytochrome P450 enzymes
CNS	Central nervous systems
DHEA	Dehydroepiandrosterone
FSH	Follicle-stimulating hormone
GWAS	Genome-wide association study
HA	Hyperandrogenism
HCG	Human choriogonadotropin
HSDs	Hydroxysteroid dehydrogenases
INSR	Insulin receptor
INS	Insulin
IRS-1	Insulin receptor substrate 1
IVF	In vitro fertilization
LHCGR	Luteinizing hormone/Choriogonadotropin receptor
LH	luteinizing hormone
MMP	Matrix metalloproteinase
PCOS	Polycystic ovary syndrome
SNPs	Single nucleotide polymorphisms
StAR	Steroidogenic acute regulatory protein
T2DM	Type 2 diabetes mellitus
UTR	Untranslated region
SHBG	Sex hormone-binding globulin
